# Chloral—A New Anæsthetic

**Published:** 1869-10

**Authors:** Geo. J. Engelmann

**Affiliations:** Stud. Med.


Chloral—A New Anæsthetic.—Berlin, June 8, 1869. Being at present engaged in the chemical laboratory attached to Virchow's Pathological Institute, it is with particular pleasure that I communicate to you an important discovery for which we are indebted to its chief, Dr. Liebreich.

Though Dr. L. has laid his discovery before the scientific men of Berlin, in both the Chemical and Medical Societies, nothing has as yet appeared in print, and the hasty account can not but be exceedingly unsatisfactory, yet I trust it will not be without interest, as being the first which crosses the Atlantic.

The researches of Dr. Liebreich have disclosed a new, and to all appearances, most valuable anæsthetic, which bids fair to rank with chloroform and morphine as one of the benefactors of suffering humanity.

Chloral ($C_2Cl_3OH$), the aldehyde of trichloretted acetic acid, has indeed been known to chemists for perhaps the last thirty years, but its valuable medicinal properties have so far been overlooked. It is a colorless fluid, of penetrating odor, but almost without taste, obtained by the action of chlorine gas upon alcohol, and is thus prepared in England on a large scale, being used for the manufacture of chloroform, as solution of caustic soda decomposes it, with production of chloroform and formate of soda $\left(\left.\begin{matrix}CCl_3\\COH\end{matrix}\right\}+\left.\begin{matrix}H\\Na\end{matrix}\right\}O=CCl_3H+\left.\begin{matrix}COH\\Na\end{matrix}\right\}O\right)$. Upon this process, the gradual decomposition of the soluble and readily absorbed chloral in the alkaline fluids of the body—this slow production of chloroform—probably depend its effects upon the system.

We may compare the action of chloral to that of chloroform inhaled in small, continued doses; in some cases a slight headache followed, apparently less than is produced by morphine. Little can, of course, as yet be said from the few cases on record, though it has been given internally with success to patients in different departments of the Charité. A solution of the hydrate in an equal quantity of water has been used—the largest quantity as yet given being 4 grammes. 4 grammes of the solution contain 2 grammes (32 grains) of the hydrate, and decomposed give 1 1-3 grammes, about 21 grains, of chloroform.

Upon animals the injection has been used with most satisfactory results; drowsiness comes on, and soon perfect stupor. The effect is mild and gradual, not the least sign of a *stadium excitatorium*, so disagreeable in chloroform. This death-like stupor was prolonged, according to the strength of the dose, as far as 18 hours; upon awakening, the animal appears in full possession of his faculties, and at once feeds.

This anæsthetic is applicable, it will appear in cases of insomnia from general suffering, mental excitement, and even in cases of insanity, where it has already been successfully tested. Though it can not be expected to supersede either chloroform or morphine, differing from both in its effects; we may confidently predict for it a wide and important field of action, and American physicians will certainly not be behind hand in giving chloral a fair test.

So much, until I shall be enabled to send you Dr. L.'s publication. Respectfully,

GEO. J. ENGELMANN, Stud. Med.

